# Facilitators and Barriers to Uptake of Community-Based Diabetes Prevention Program Among Multi-Ethnic Asian Patients With Prediabetes

**DOI:** 10.3389/fendo.2022.816385

**Published:** 2022-02-28

**Authors:** Sungwon Yoon, Sharon Wee, Dionne H. F. Loh, Yong Mong Bee, Julian Thumboo

**Affiliations:** ^1^Health Services and Systems Research, Duke-NUS Medical School, Singapore, Singapore; ^2^Centre for Population Health Research and Implementation, SingHealth Regional Health System, Singapore, Singapore; ^3^Department of Endocrinology, Singapore General Hospital, Singapore, Singapore; ^4^Department of Rheumatology and Immunology, Singapore General Hospital, Singapore, Singapore

**Keywords:** diabetes prevention, social and behavioral strategies, health promotion, Asian patients, prediabetes

## Abstract

**Objective:**

This study aimed to identify facilitators and barriers to the uptake of a community-based diabetes prevention program (DPP) from the perspectives of decliners with prediabetes in a multi-ethnic Asian community.

**Methods:**

Semi-structured interviews were conducted with 29 individuals with prediabetes who declined participation in a large community-based diabetes prevention program in Singapore. Thematic analysis was undertaken to identify themes, which were subsequently mapped onto the Capacity-Opportunity-Motivation and Behavior model (COM-B).

**Results:**

We identified 16 key themes under three COM-B domains. Health status at the time of invitation, perceived ability of self-management, understanding of prediabetes condition and/or the program intention (*Capability*) were important determinants. Family commitment had the strong potential to enable or hinder physical and social *Opportunity* related to participation. Many participants desired involvement of physician as part of program invitation and component. Fear of exacerbation coupled with an automatic aversion for suffering influenced *Motivation* for participation.

**Conclusion:**

Identifying facilitators and barriers embedded in the COM-B will assist systematic program modifications to increase participation of individuals with prediabetes. How information about modifiable risk factors is communicated by physicians at the point of diagnosis and program introduction is key to participation. Co-locating programs with family activity, development of mHealth, readiness assessment, and tailored explanation of program purpose may increase participation. These findings will be used to guide future national interventions in the community to ensure successful implementation.

## Introduction

Prediabetes is defined as an elevation of plasma glucose above the normal range but below the diagnostic threshold for diabetes. Globally, the prevalence of prediabetes is growing. It is estimated that more than 470 million people worldwide will have prediabetes in 2030 (1). In a multi-ethnic city-state of Singapore, one in seven adults aged 18 to 69 has prediabetes (2).

It is well established that prediabetes is associated with an increased risk of progression to diabetes and increased mortality even before the onset of diabetes (3–5). Evidence suggests that the total economic cost for patients who develop diabetes compared to those who do not transition from prediabetes to diabetes is found to be 42% higher (6). Therefore, early intervention to prevent and delay the progression could substantially reduce healthcare costs resulting from diabetes and its complications and improve health and quality of life for a large number of populations.

There is compelling evidence that structured and intensive lifestyle interventions and metformin are effective in reducing the onset of diabetes among people with prediabetes (7–11). In light of the rising disease burden resulting from diabetes, many countries adopted lifestyle intervention programs such as the Diabetes Prevention Program (DPP) to reduce the progression from prediabetes to diabetes through population-wide approaches (12–16). Similar to this trend, Singapore has adopted a large scale community-based DPP. Such interventions are only effective if uptake of the targeted population is high. Unfortunately, participation rates of the program have been less than optimal. According to ongoing intervention data, two-thirds of eligible participants were found to be unwilling to participate (17).

Studies suggested that several factors hinder the successful adoption of DPPs for persons with prediabetes. They included a lack of awareness of having prediabetes, time commitment, costs of participation, poor knowledge of prevention programs, low self-efficacy, perceived health status, and limited support from social network (18–22). However, the bulk of studies tended to utilize data from either a subset of participants who had already completed interventions (and thus may have a greater motivation to change) or a pool of ‘at-risk’ individuals who had *never* been actively offered preventive interventions. Conclusions based upon these groups may not reflect the true motivations and behaviors of individuals who are *eligible* and *offered* to participate. In addition, currently available evidence is primarily based on studies conducted within a Western country. It is therefore important to elucidate culturally pertinent accounts of factors that may enable or hamper participation among the Asian population with prediabetes.

To fill these gaps, we performed a theory-informed identification of facilitators and barriers to participation in a community-based DPP from the perspectives of decliners with prediabetes in a multi-ethnic Asian community, using the Capacity, Opportunity, Motivation and Behavior model (COM-B) (23). To avoid merely capturing idealized justifications for non-participation, we aimed to elicit decliners’ experience of program invitations, how the decision to decline was reached, and the factors that influenced their deliberations, an important dimension that has not been paid much attention to in the existing literature.

## Materials and Methods

### Conceptual Framework

To explore behavior-oriented accounts of factors that influenced the deliberation of non-participation, this study employed the Behavior Change Wheel as a theoretical framework. The Behavior Change Wheel is a behavioral health framework developed by Michie and colleagues deriving from 33 commonly used behavioral theories. It is underpinned by the COM-B model, which postulates that for behavior change to occur, three conditions must be met: Capability, Opportunity, and Motivation (23). Capability refers to the individual’s capacity to perform the behavior and includes both physical and psychological capability. Opportunity refers to factors external to the individual that enable or hinder the behavior and includes social and physical opportunity. Lastly, motivation refers to beliefs, emotions, and impulses that direct behavior and includes reflective and automatic motivation. Reflective motivation is motivation that involves conscious thought processes while automatic motivation involves habitual, instinctive and affective processes. The COM-B model offers a framework to systematically develop interventions for behavior change. The model was particularly useful for this study as it enabled us to systematically identify a range of motivational, behavioral and systemic factors that may affect deliberations on program participation. This information would improve understanding of preferences for various aspects of program options and inform future nation-wide implementation of the community-based DPP *via* key modifications to improve feasibility and dissemination.

### Study Design and Sample

We used a qualitative research method to gain a systematic understanding of facilitators and barriers of non-participation in a community-based DPP in Singapore. Respondents were those who had rejected participation in the Pre-Diabetes Interventions for Continued Tracking to Ease-out Diabetes (Pre-DICTED) Program (henceforth Pre-DICTED), a large-scale government-sponsored 5-year open-label 1:1 randomized controlled trial that aims to reduce the risk of type 2 diabetes over 3 years among individuals clinically diagnosed with prediabetes. The core intervention phase of the Pre-DICTED consists of twice-weekly lifestyle classes (nutrition workshops, exercise sessions, and a goal-setting workshop) for 6 weeks, followed by 6 weeks of self-directed lifestyle modifications. There is an add-on metformin prescription if glucose control remains poor after 6 months of the intervention. Further details of the program can be found in the published protocol (17).

For the current study, respondents were informed of the study after they had declined participation in the Pre-DICTED and agreed to have their contact details released to the study team. They were approached *via* phone call, and the study purpose and scope of confidential and anonymous participation were explained. Upon verbal agreement of participation, an interview date, time, and venue were arranged. Informed written consent was obtained prior to the interview. Patients were selected purposively according to age, gender, and ethnicity to capture the richness of a broad range of views and experiences. A total of 38 patients were contacted, and 29 individuals agreed to participate in the interviews (socio-demographic details of participants is provided in **Additional File 1**). Major reasons for decline included disinterest and being too busy.

### Data Collection

We developed a semi-structured interview guide with open-ended questions to solicit the respondents’ experience with the program invitation, risk perceptions of diabetes, context surrounding decision making for non-participation, and preferences for program components. The interview guide was pilot tested with 5 respondents and revised. Two study team members trained in qualitative research conducted one-to-one interviews. As program decliners are arguably a hard-to-reach group, one-to-one interviews were chosen to improve participation in the study and to reduce the risk of socially desirable responses for the decline in program participation in the context of a group discussion. Each interview lasted approximately 45 to 60 minutes and was audio-recorded with the permission of the respondents. The study was approved by SingHealth Institutional Review Board (CIRB ref 2017/2597).

### Data Analysis

All interviews were audio-recorded and transcribed verbatim. Thematic Analysis was performed based on constant comparison with grounded theory (24). The analytic process involved immersion in the data, coding, repeated sorting, and comparison. Each transcript was open-coded line by line to create code components. Each component was compared with other components to ensure that they were mutually exclusive. Following iterative comparisons of components, they were grouped into subthemes and further abstracted to form broader themes. Themes were then continually reviewed, refined, and classified until no new themes emerged from the data while accounting for deviations. Themes were subsequently mapped to the components of the COM-B model to systematically identify barriers and facilitators for Pre-DICTED participation (25). All transcripts were independently coded by two coders (DL, SW). Discrepancies were resolved through an iterative consensus process involving the 3^rd^ researcher (SY). This allowed for inter-coder clarification of themes and sub-themes mapped against COM-B components, thus enhancing validity and reliability. NVivo 12 was used for data management and coding. For rigor and transparency, we anchored our methodology according to the Consolidated Criteria for Reporting Qualitative Research (COREQ) checklist (26) (see **Additional File 2**).

## Results

Data saturation was reached at 25 interviews, and four more interviews were conducted to ensure if any new themes emerged. Respondents’ age ranged from 28 to 64 with the mean age of 51 years. The majority were married (72%) and attained secondary education (76%). Approximately 58% were female and Chinese (**Table 1**). Several important themes emerged from the interviews. **Table 2** lists illustrative quotes categorized according to the COM-B components: Opportunity, Capability, and Motivation.

**Table 1:** Respondent characteristics (N = 29).

	N (%)	
Age (year)		
Mean (SD)	51.4	(10.6)
Range	28 - 64	
Gender		
Male	12	(41.4)
Female	17	(58.6)
Ethnicity		
Chinese	17	(58.6)
Malay	9	(31.0)
Indian	3	(10.3)
Education		
None/Primary	7	(24.1)
Secondary	12	(41.4)
Tertiary or above	10	(34.5)
Marital Status		
Single/Never married	4	(13.8)
Married	21	(72.4)
Divorced/Widowed	4	(13.8)
Having a Family Doctor		
Yes	12	(41.4)
No	17	(58.6)

**Table 2:** Facilitators and barriers to uptake of diabetes prevention program.

Component	Barrier	Facilitator	Illustrative quotes
Physical capability	Physical weariness		I cannot do vigorous exercise because my bones are too weak and feeble to follow the kind of exercise. I had a fracture before. I don’t think I will be able to continue (14)
			I got oral lichen planus, so my muscles and my joints, they do ache almost every day. So sometimes I don’t feel well to bring myself to exercise. I generally feel under the weather or have fatigue. I simply don’t have the energy. This is one thing I have to battle [with] very frequently (8).
	Ability of self-management		Then the (HbA1c) result came out and it is the same. Few years ago, also 0.1, 0.2, now also the same. So that means to me, the result, few years back and now, there is no increase. So, I think that through my lifestyle I did something right (3).
			I told the person on the phone that I KNOW what to do to bring down my sugar level, and I know what is needed to be done, so she said that it’s good that I know what is needed to be done, and I declined the offer of taking up the program (24).
Psychological capability	Lack of awareness of prediabetes or Pre-DICTED due to asymptomatic nature of prediabetes		I do not feel much, other than being overweight. I do not feel so-called discomfort so I don’t see the need (27).
			I’m pre-diabetic, but I feel nothing. Just a bit worried (about) the next time (when) the doctor asks me to go check-up [laughs], will I be confirmed (to have diabetes), then I have to watch out for my diet then. So for now, as long as it’s not here yet (diabetes), then I still drink coffee as usual with sugar, but maybe once I have (diabetes), then I have to control – no more sugar already [laughs] (7).
		Recognition of prediabetes being reversible with lifestyle changes	Prediabetes, this type can be controlled by myself, through diet. That means your lifestyle. If your lifestyle and your weight can everything in a good condition I think this one the number (referring to HbA1c) will go down (3).
			The best part is you have to take care of your diet, because there is hope that you can stop this … If they do not take care, then it would come to a stage where they would definitely get diabetes (19).
		Understanding of progression which can lead to complications and affect livelihood	When I was sick, there were a lot of problems. I went to see the doctor at times, but the company [I worked previously] was a little unhappy. I feel that diabetes is more severe, because there would be a lot of things that they need to check, kidneys, eyes, and a whole lot of problems. The effect on lives won’t be great unless one has the ability to apply for welfare (6)
			It is a very dreadful thought that you have to pop more pills … Definitely the medical cost as well. And you don’t know what complication it will lead you to. Because once the person becomes diabetic, it’s a very suicidal thing in a way. But if you look at the positive side of it, if you start taking positive actions, a diabetic patient can live a good quality life (8).
Physical opportunity	Program components -inconvenient timing and location -not wanting to take medication -availability of other similar programs		My schedule,… I am working Wednesday night and Saturday morning (which are the days that Pre-DICTED is running) (23)
			Because I am doing night shift. I am doing permanent night and I am having my own business that is why I cannot commit the time to travel (19).
			The location, I don’t know how to go there. This is the problem. If there is an end-to-end bus, it’s okay for me. MRT also like that, if end-to-end, then it’s also okay. But need to change the MRT, go to Purple Line, Red line, alamak [Malay; a form of exclamation]! So, that’s why it’s a problem! (21)
			I want to change my life first, I don’t want to do the medication first … Because once you take the medication, you must take it every day. At least if you try others like exercise, you will try first to prevent that … About the safety (of the medication), I am very worried about that (22).
			Actually, there are a lot of free exercises around, so I am not very willing to fork out money [deposit of $20 that would be refunded] for exercise (28).
		Program components -preference for group activity -weight loss as a program outcome -self-management app	The group participant can see how each and everyone is doing. Like the percentage of fat dropped, the muscles mass increase, or the weight loss something like that. So, it becomes peer motivation. So probably it will help (8).
			Weight loss is definitely the main motivation. If I can drop down to 75, that’s a blessing in disguise (13).
			Okay, let’s say if they have some videos they want me to look at, maybe I can do that, while online. Because if they have some program to reduce the risk of getting diabetes … then I can watch at my own time. I can also follow through at home. Maybe something like that will be better (10).
			I think a mobile app can serve as a reminder. Because with the application, sometimes when we want to eat things, we are reminded not to do so and how to restrain. I feel that if they send this message, it is good as it gives us a reminder (6).
Social opportunity		Desire to have healthcare professionals involved in the program	There won’t be any meeting with doctor face to face. So, I thought probably this would not fit me very well because I do exercise on my own … doctors are the subject matter expert. They can give medical advice and tell you what you should do and you take their advice seriously (13)
			In the midst of the program, if we encounter any problems, health (related) people or at least the doctor might be able to advise us (29).
	Heavy family commitments		I told them that I would consider this with my husband first then I would let you know because actually I got a kid here so I can’t just like travel here and there … my husband just says, ‘it’s a bit difficult, because you got kid so you cannot go here and there all that.’ (9)
			…only Saturday classes and only one weekday night class [referring to Pre-DICTED schedule]. So, during that time, I do volunteer work … then I’m handling my boy who is this year PSLE [national exam]. So, it’s a bit tight schedule for me. In terms of my time, I can’t participate due to my kids… (18).
		Wishing to continue to financially support family and not to be a burden to them	If you become serious then amputation all these, it would affect your family life. I mean, we always tell ourselves, if we’re sick, never mind, but that will affect my family … I must be able to take care of myself. I do not have to rely on anybody. My own family members, I need to take care, for their sake (14).
			She [wife] is the one that motivates. I see her I pity her … If anything happens to me, it will be a burden. So, I have to be strong and I need to change (12).
	Absence of recommendation by primary care physician; insufficient advice given by healthcare professionals		Although (polyclinics) have so many [lifestyle] programs, [my] doctor did not ask me personally to go for it, maybe it is not their job … Once (the doctor had) the blood test results, that is the time the doctor (should) give me the brochure to tell me about a suitable program, and strongly encourage me to go rather than, “you take this medicine…” (9)
			They didn’t advise me on what diet I should take. Just saying ‘basically you have prediabetes, eat like you’re a diabetic patient’. They never tell you what things to watch out and what to avoid … Not very forthcoming (11)
		Helpful communication with healthcare professionals	The doctor advised me to go counseling. He tells me what I can eat, what I cannot eat, how to control. Find out about my history, about my background then advised me to do one-week three times exercise … So, I follow accordingly (15)
			The doctor say(s) that it doesn’t mean that you reduce the sugar level, then that’s it. You still need to exercise to be more assured that this pre-diabetes can be reversed, so I take his advice seriously. I mean, whatever he tells me, I think, is for my own good (19).
Reflective motivation		Fear of exacerbation in future due to inaction	Mentally, I think I’ll be very worried every now and then. ‘how’s my sugar level today, you know. Did I eat something wrong?’ You will be more afraid to fall, because you are scared to get your leg cut and then the wound cannot be healed (26).
			If I keep taking sick leave, maybe due to illness, it affects my performance. let’s say in the time where company needs to downsize, then probably you will be the first to go because of your contributions compared to others, you are always not in the office and feeling tired as well (27)
		Desire to maintain independence and mobility	Hope that I can age more gracefully, and I can be independent. I don’t have to be that sick and depend on somebody else. I don’t want to lose my independence (9)
			Because when I travel, I look for food, looking at scenery and all those. Can you imagine you have diabetes and then you have to amputate your foot and then you cannot go and (sight-)see? (3)
Automatic motivation	Lack of interest		I [laughs lightly] said I’m not interested [laughs] because maybe I don’t want to travel so far (8).
			Yeah, I heard about all these types of things but to me, after the blood test, the numbers that I have are just 0.1 or 0.2. So I just let it go. I do not want to go further to know more about it. Unless mine is very high then maybe I will (4).
		Emotions evoked by witnessing experience of significant others with diabetes (i.e., suffering)	Seeing her suffer every day when we go to the dialysis center when they take out the needle, the blood will spill on the floor. One thing about diabetes is, if you do not take your medicine, either any part of your body will be amputated. If you take your medicine also your kidney cannot take it for the long run (24).
			…afraid because my aunt had her toe cut then she passed on. So when I think of her, I think that diabetes can kill, so how to prevent, how to control. That would make me more interested to join the program (5).

### Physical Capability

Pre-existing conditions such as ongoing inflammatory conditions and arthritis (e.g., gout) were reported as a common factor given by respondents that impeded physical capability of participating in the program. Despite generally positive attitudes towards the Pre-DICTED, accounts of these individuals centered on the physical inability to participate. Physical weariness was cited more predominantly among older participants. Notably, a small number of respondents maintained that they had the necessary *capability* to make lifestyle changes on their own and hence did not feel the need to participate in the program.

### Psychological Capability

The main challenge surrounding psychological capability stemmed from respondents’ lack of understanding and knowledge of either the Pre-DICTED or the prediabetes condition. Many reported opening the invitation letter and briefly glancing through the leaflet, while others described reading the leaflet in detail. Most also remembered that they had been contacted by telephone to join “some workout, fitness program” (participant 10). However, several respondents expressed confusion over what the program was intended for. As one respondent recalled, the information contained in the invitation letter and subsequent follow-up call did not help her comprehend “what was going on” (participant 06) with the program. They decided not to participate, perceiving that they did not understand the benefits of the program.

Knowledge of prediabetes seems to be both a barrier and an enabler for program participation. Since prediabetes is asymptomatic, some respondents did not feel the pressing need to take action to prevent diabetes. Despite the acknowledgment of being at risk, some described their condition not as severe as people with diabetes and therefore in less need of “intervention”. Other respondents labelled themselves as “normal” rather than being overweight. On the other end of the spectrum, respondents recognized that prediabetes is a reversible condition with lifestyle changes. Having an awareness of the health consequence of prediabetes was an important step towards considering the Pre-DICTED for those whose *physical opportunity* was the main barrier, as described in the next sub-section. For this category of respondents, there was a strong desire to prevent disease progression, and this could act as an enabler to participate in the program.

### Physical Opportunity

We found that logistical aspects of the Pre-DICTED was the most commonly reported factor limiting physical opportunity to participate. For example, respondents found the location of the program inconvenient, involving long traveling time away from home. Besides physical distance, timing of sessions, which typically takes place on workdays, was reported to be a key consideration alongside other inconvenience. Some respondents described that they were working multiple jobs and hence could not physically find time to attend the program sessions. For example, younger respondents reported that it was harder to commit to a single fixed slot as their shift schedules varied week to week and rarely conformed to typical 9-6 work hours. In such cases, declination was quick with very little deliberation. The program component also emerged as something that impeded physical opportunity. For example, some respondents thought that metformin was a compulsory component and hence declined participation as their preference was for lifestyle changes alone as opposed to the combination of medication and lifestyle intervention. Another important factor that hindered participation was perceived cost. As one participant mentioned, there are “many *free* workout programs available in the community” and hence the Pre-DICTED was not something that is worth “forking out money (i.e., a deposit of $20 to be refunded at the end of the trial) just for exercise” (participant 29). The Pre-DICTED decliners had a general tendency to have little willingness to participate if financial commitment would be required.

Despite these barriers, the program had many components that seemed to be attractive to the respondents and hence would act as an enabler for participation. For example, some expressed an interest in the program’s group-based activity as they found peer support to be helpful for motivating them to continue engaging in the program. However, a minority of respondents preferred one-to-one sessions. Weight loss will be an important outcome for respondents to look forward to if they join the program as many explicitly stated that they would not consider if the focus of the program is not on weight loss. Across interviews, it became evident that clinical benefits of the program such as reversing high blood glucose, appear to be secondary to the program participation when compared with more tangible and perceivable outcomes such as weight loss.

### Social Opportunity

Running through the interviews was the role of *interpersonal influences* that limited respondents’ opportunity to engage in the program. The commonly reported barriers to social opportunity included family and healthcare professionals. Many respondents, particularly female decliners, recounted how difficult it was to take up the program when they had to look after young children. Respondents also expressed unhappiness with the healthcare experience when providers were not forthcoming with adequate explanations on their health conditions. Some felt very strongly that besides prescribing medicines, doctors in primary public clinics rarely encouraged patients to take part in any lifestyle prevention program during the consultation. They expressed a desire for more information to be presented in layman’s term with less medical jargon.

At the same time, family and healthcare providers were cited as enablers for participation in the program. For example, having a good relationship with and trust in healthcare professionals played a key role in respondents’ deliberation on participation. A handful of respondents appreciated the advice given by their healthcare providers, including detailed diet and physical activity advice and explanation on progression from prediabetes to diabetes. It was commonly maintained that if family doctors had recommended a prevention program, it would certainly have considered participating in the Pre-DICTED. Importantly, there were frequent references to family responsibilities as an enabler among our respondents. Almost all respondents expressed a desire to maintain their health because they wished to continue to work and provide their family with financial support and stability. Most of the accounts seemed to reflect on respondents’ strong desire not wanting to become a burden to family if their prediabetes condition is ever worsened. Some did go on to imagine the difficult situation that could arise.

### Reflective Motivation

Barriers were also described as relating to *reflective motivation*. Respondents who reflected on their susceptibility to developing diabetes and its complications tended to have greater motivation towards making lifestyle changes and hence participation in the program. In most cases, respondents expressed a desire to maintain their independence and mobility, and this would act as an enabler. Awareness of psychological consequences of diabetes was present in many interviews, and this emerged as a powerful theme. Respondents commonly stated that having diabetes can be mentally stressful. Some often vividly portrayed the fear of falls as a result of poor wound healing, concerns about amputation, and the stress that declining work performance would lead to retrenchment.

### Automatic Motivation

Despite differing levels of capability and opportunity, respondents who reported empathy when they witnessed close family members’ suffering from diabetic complications appeared to be more receptive to participation in the program. For example, one respondent described how painful it was to observe her mother going through daily dialysis, alluding to the importance of prevention, while another respondent witnessed her aunt’s passing after amputation that encouraged him to consider participation in the program. Nonetheless, emotional reactions were not common to every participant. Some asserted that having diabetes did not affect the family member’s lifestyle, and hence diabetes may not be a serious problem. There were a couple of respondents who simply believed that prevention programs were not of interest to them.

## Discussion

**Figure 1:**
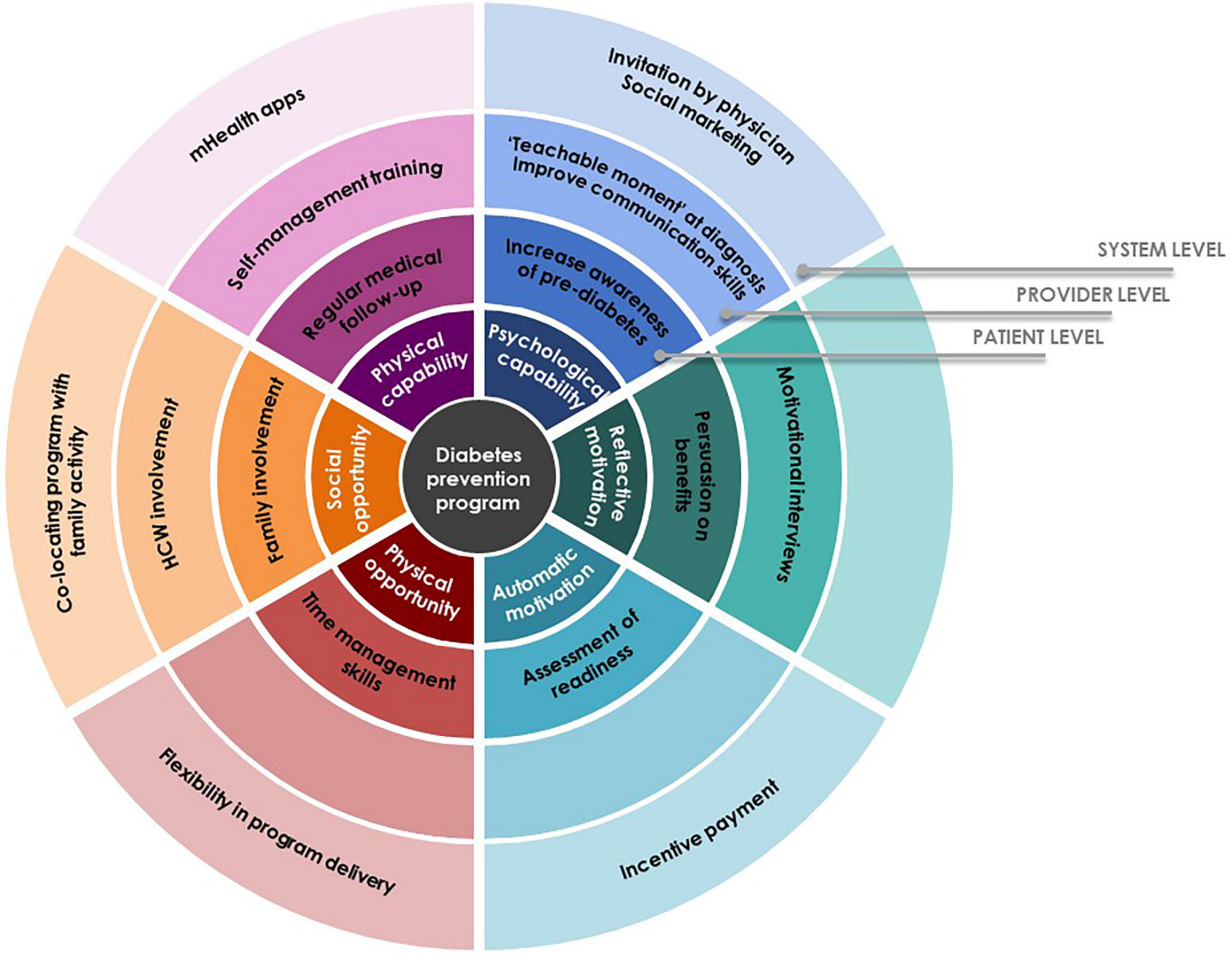
Potential intervention strategies.

This study sought to identify factors that enable or hamper participation in community-based diabetes prevention program among decliners with prediabetes. Based on our findings, we have developed potential intervention strategies that should be considered for implementation from patient level through system level to better facilitate uptake around the six spokes of the Behavior Change Wheel (**Figure 1**).

Our finding shows that decisions of individuals with prediabetes hinged on several *physical and psychological capabilities*: health status at the time of the invitation; perceived ability of self-management; and understanding of prediabetes condition and the Pre-DICTED. Understanding the reversible nature of prediabetes appeared to influence people to make behavior changes. However, limited understanding and appreciation of the program purpose and benefits at the point of invitation acted as a hindrance to participation. It is therefore important to adopt an approach designed to address key issues related to *capabilities*, such as improving communication skills of program staff who recruit participants and more attention to how messages about the program and diabetes prevention are conveyed. Literature suggests that diagnosis of prediabetes can be a ‘teachable moment’ (18, 27–30) and presents an opportune time to introduce preventive interventions to patients. The attitude of and messages conveyed by healthcare professionals were also found to affect an individual’s views of the gravity of the condition and hence his/her commitment to making changes (18, 31, 32). Indeed, our respondents expressed a desire for recommendation or referral by primary care physicians to consider participation, and this finding resonates with prior research (19). Yet, a lack of awareness of preventive programs can impede healthcare providers from confidently referring patients (33). At a system level, a social marketing campaign targeted at primary care can be considered to increase awareness of programs among healthcare providers and patients (33, 34). While teachable moments within the context of a diagnosis can present valuable opportunities for healthcare professionals to introduce a diabetes prevention program, findings presented here and others showed that at-risk individuals perceived different degrees of severity and that family experiences with diabetes could influence risk perceptions (35, 36). Therefore, communication could be tailored to personal experience to maximize the benefits of teachable moment. On the other end of the spectrum, several respondents who felt their self-management efforts were sufficient did not see a need for the Pre-DICTED and this is similar to a prior study (30). However, maintaining motivation to persist with behavior changes can be challenging as shown in the literature (28, 37, 38). Thus, support should be extended to these decliners through more information and avenues for self-help at the level of patients. A randomized controlled trial conducted among decliners of DPP found that a mobile health (mHealth) intervention was acceptable, particularly for those who demonstrated a motivation to undertake lifestyle changes (39, 40). Therefore, mHealth would be a good complement to the on-site structured program to increase self-efficacy and improve responsiveness to a range of individual needs.

Our findings underline the importance of recognizing modifiable *physical and social opportunities* leading eligible individuals to opt-out of the program. Similar to prior literature (30, 33), the most commonly cited factors for non-participation were *physical* location and scheduling of the program. In countries such as the US and UK, many DPPs are outsourced, which allows for flexibility in program delivery (e.g. co-locating programs with other services such as gym and childminding or near workplaces) (29, 33, 34, 41–43). In a study by Van Name et al, a modified DPP conducted in the cafeteria or classrooms of a school, while offering a parallel program of play-based physical activity for children at the school, demonstrated not only the feasibility and effectiveness, but it was also well received by participants (37). In our study, a heavy family commitment was a common reason among female respondents for program rejection (44). A similar model of delivery, for example, co-locating the Pre-DICTED with family activities, should be considered for those who have interest but lack *physical and social opportunities*. Having family members join group activities has shown to improve glycemic control, diabetes knowledge, self-efficacy and quality of life (37, 45–47). While family can be both a barrier and facilitator for lifestyle changes, their influence is substantial, especially in Asian cultures, and hence family should be included as agents of change in any proposed intervention (44, 48–55). In addition to program-related factors, inadequate comprehension of requisite program components (e.g., metformin) were important determinants hindering participation. To encourage individuals with prediabetes to fully engage, program components should be clearly explained and adapted to the needs of target participants.

Our study identified some *motivational* factors that act as an enabler for improving participation. They included fear of exacerbation due to inaction, desire for maintaining independence, coupled with an *automatic* aversion for suffering as a result of observations from experiences of significant others with diabetes. Not wanting to become a burden to the family financially or with care responsibilities featured prominently in our study and was a sentiment especially pertinent in collectivist cultures (47, 52). While fear of disease severity as informed by personal observation within social networks appears to motivate some to alter their attitudes, persuasion of participation based on one’s belief about severity alone may not be fully effective (32). Evidence suggests that effective program introduction can help patients to contextualize the program and make it personally meaningful to motivate their participation (32, 42, 56). To this end, motivational interviewing (MI) can be considered at the program introduction to foster participation. A brief assessment of intrinsic motivations would allow program staff involved in recruitment to identify those with low interest in behavior changes (*automatic motivation*) and use that to tailor the conversation to foster more autonomous forms of motivation. A systematic review shows that diabetes prevention programs involving MI facilitate autonomous motivation which is then translated into improved physical activity and weight loss (57). Most empirical studies have dealt with the efficacy of MI in the treatment of lifestyle problems and diseases alone. Future research could usefully explore how brief MI-based invitation can improve uptake of disease prevention programs. Financial incentives might be also considered as a mechanism to improve engagement in at-risk individuals who are not ready to change behaviors. A community-based randomized controlled trial found that participation in a DPP was significantly higher for participants who received incentives compared to those who did not (58) and the intervention remained cost-effective even with the provision of direct financial incentives (59).

### Limitations

The present study adds to the scant literature on theory-informed factors that may influence non-participation in DPP. To our knowledge, this is one of the few studies that explores potentially modifiable factors that may influence the decision to decline in the prediabetes program, using a structured behavioral science framework. However, findings from this study should be considered in light of a few limitations. The voluntary nature of participation impacted the recruitment process, which may have generated selection bias. Those respondents who chose to participate in this qualitative study may have felt more positive about the Pre-DICTED than those who did not respond to our invitation. Some interviews took place months after the initial decline in the Pre-DICTED, which may have limited respondents’ ability to recall details and hence the deliberation process. Although we recruited a diverse range of respondents, it was not possible to explore age or gender-specific accounts of enablers and barriers, although no distinct difference was observed.

## Conclusions

This study underlines that diagnosis of prediabetes may be a teachable moment that presents a salient window of opportunity to improve program uptake. How information about modifiable risk factors is presented and by whom can be an important determinant of participation. This has implications for patient education and involvement of physician, particularly at the point of program introduction. For program structure and content, considerations should be given to more flexibility (e.g., complementary mHealth apps, co-locating programs with family activity) to optimize the program thereby increasing participation. These findings will be used to guide future national interventions in the community to ensure successful implementation.

## Data Availability Statement

The original contributions presented in the study are included in the article/**Supplementary Material**. Further inquiries can be directed to the corresponding author.

## Ethics Statement

The studies involving human participants were reviewed and approved by SingHealth Centralised Institutional Review Board. The patients/participants provided their written informed consent to participate in this study.

## Author Contributions

SY, YB, and JT conceptualized the study. SY designed the question guide. SW and DL conducted some of the interviews. DL, SW and SY performed the analysis and interpretation of data. All authors read and approved the final manuscript.

## Funding

The Pre-DICTED programme (The Pre-Diabetes Interventions and Continued Tracking to Ease-out Diabetes) was supported by Ministry of Health, Singapore. This work was supported by the National Medical Research Council (NMRC) through the SingHealth PULSES centre grant NMRC/CG/027/2017. The funding body had no role in the study design, collection, analysis, and interpretation of data and manuscript writing.

## Conflict of Interest

The authors declare that the research was conducted in the absence of any commercial or financial relationships that could be construed as a potential conflict of interest.

## Publisher’s Note

All claims expressed in this article are solely those of the authors and do not necessarily represent those of their affiliated organizations, or those of the publisher, the editors and the reviewers. Any product that may be evaluated in this article, or claim that may be made by its manufacturer, is not guaranteed or endorsed by the publisher.
