# Supplementary material for: Facilitators and Barriers to Uptake of Community-Based Diabetes Prevention Program Among Multi-Ethnic Asian Patients With Prediabetes
Source: Front Endocrinol (Lausanne). 2022 Feb 28;13:816385. doi: 10.3389/fendo.2022.816385 (PMC8919042; doi:10.3389/fendo.2022.816385)
Supplement: Supplementary file 1 [file DataSheet_1.pdf]

Interview Guide

Facilitators and barriers to uptake of community-based diabetes prevention program among multi-ethnic Asian patients with prediabetes

1. Can you tell me a bit about your condition? How did you learn that you have prediabetes?

2. What is your understanding of prediabetes? What are your thoughts on your risk of developing type 2 diabetes?

3. What are some ways you have tried to change your lifestyle behaviors (e.g., physical activity, diet and personal habits)? What prompted those changes? What things have been helpful?

4. Tell me about things you tried but were unsuccessful. What problems did you have? What things have affected the unsuccessful attempt?

5. How important is it for you to make lifestyle changes to prevent diabetes? What would help you to stay motivated to follow a lifestyle that can prevent diabetes?

6. How easy or difficult to make lifestyle changes for you?

7. Can you tell me about your experience regarding the invitation to participate in the Pre-DICTED program (invitation letter, phone call)? How did you learn about the program? What did the program staff tell you and what was your response?

8. How did you come to a decision to not participate in the Pre-DICTED program? Can you walk me through your deliberation process?

9. What things influenced your decision to decline the invitation? Can you elaborate? *Prompt - personal/social (e.g., your interest, motivation, feelings and circumstances, your physical or mental stamina or limitations, support from family and friends), program related (e.g., content, benefits, timing, location, finance, support, degree of autonomy) or barriers in behavior changes mentioned earlier by the participants.* Which one(s) were more important than others and why?

10. Moving forward, how do you think the program invitation can be improved to foster uptake of people with prediabetes?

11. In the future, what factors might affect your willingness to participate in similar community-based diabetes prevention programs? What information would be useful?

Is there anything else you would like to add on the topics we have discussed today?
