# Supplementary material for: Facilitators and Barriers to Uptake of Community-Based Diabetes Prevention Program Among Multi-Ethnic Asian Patients With Prediabetes
Source: Front Endocrinol (Lausanne). 2022 Feb 28;13:816385. doi: 10.3389/fendo.2022.816385 (PMC8919042; doi:10.3389/fendo.2022.816385)
Supplement: Supplementary file 2 [file DataSheet_2.pdf]

Table S1. Demographic details of respondents (N=29)

Participant No	Gender	Age	Ethnicity	Highest level of education	Marital status	Having a regular family doctor
01	Female	63	Chinese	Primary	Married	No
02	Male	33	Chinese	Tertiary or above	Married	Yes
03	Female	63	Chinese	Secondary	Divorced	Yes
04	Male	54	Malay	Secondary	Married	No
05	Female	49	Malay	Tertiary or above	Married	No
06	Female	55	Chinese	No formal qualifications	Single/ Never married	No
07	Female	64	Chinese	Secondary	Married	No
08	Female	48	Chinese	Tertiary or above	Single/ Never married	Yes
09	Female	42	Indian	Secondary	Married	No
10	Female	37	Indian	Tertiary or above	Married	No
11	Female	62	Chinese	Secondary	Married	No
12	Male	55	Malay	Secondary	Married	Yes
13	Male	59	Chinese	Tertiary or above	Married	No
14	Male	63	Chinese	Secondary	Married	No
15	Male	63	Chinese	Primary	Married	Yes
16	Male	60	Malay	Secondary	Married	Yes
17	Male	47	Malay	Primary	Married	No
18	Female	37	Malay	Secondary	Married	Yes
19	Male	34	Indian	Tertiary or above	Divorced	No
20	Female	57	Chinese	Secondary	Married	Yes
21	Female	52	Malay	Primary	Divorced	No
22	Male	61	Chinese	Primary	Married	No
23	Male	45	Chinese	Tertiary or above	Single/Never married	Yes
24	Male	28	Malay	Tertiary or above	Married	Yes
25	Female	60	Chinese	Primary	Married	No
26	Female	45	Malay	Secondary	Married	No

27	Female	54	Chinese	Secondary	Married	No
28	Female	52	Chinese	Tertiary or above	Widowed	Yes
29	Female	44	Chinese	Tertiary or above	Single/Never married	Yes

Table S2. Consolidated criteria for reporting qualitative research (COREQ): 32-item checklist

**Item number**	**Guide questions**	**Reported on**
**Domain 1: Research team and reflexivity**		
Personal Characteristics		
1. Interviewer/facilitator	Which author/s conducted the interview or focus group?	In Methods
2. Credentials	What were the researcher's credentials? E.g. PhD, MD	PhD, M. Soc. Sci and B. Eng.
3. Occupation	What was their occupation at the time of the study?	Academic faculty in the University and staff at a medical research centre
4. Gender	Was the researcher male or female?	Females
5. Experience and training	What experience or training did the researcher have?	Training in health services and public health
Relationship with participants		
6. Relationship established	Was a relationship established prior to study commencement?	No relationship was established prior to study commencement.
7. Participant knowledge of the interviewer	What did the participants know about the researcher? e.g. personal goals, reasons for doing the research	Participants were aware of the primary purpose of the study (in data collection)
8. Interviewer characteristics	What characteristics were reported about the inter viewer/facilitator? e.g. Bias, assumptions, reasons and interests in the research topic	Interviewers have experience in conducting qualitative interviews (in data collection).
**Domain 2: study design**		
Theoretical framework		
9. Methodological orientation and Theory	What methodological orientation was stated to underpin the study? e.g. grounded theory, discourse analysis, ethnography, phenomenology, content analysis	Inductive (grounded theory) and deductive (COM-B model) (in data analysis)
Participant selection		
10. Sampling	How were participants selected? e.g. purposive, convenience, consecutive, snowball	Purposive sampling was employed (in Methods)
11. Method of approach	How were participants approached? e.g. face-to-face, telephone, mail, email	Via phone call (in Methods)

12. Sample size	How many participants were in the study?	29 (in Methods)
13. Non-participation	How many people refused to participate or dropped out? Reasons?	9 because they were busy or disinterested (in Methods)
Setting		
14. Setting of data collection	Where was the data collected? e.g. home, clinic, workplace	At home and common spaces (e.g. void deck, food outlets).
15. Presence of non-participants	Was anyone else present besides the participants and researchers?	A couple of participants had young kids in attendance.
16. Description of sample	What are the important characteristics of the sample? e.g. demographic data, date	Characteristics of the sample were described in Results and Table 1.
Data collection		
17. Interview guide	Were questions, prompts, guides provided by the authors? Was it pilot tested?	In Methods
18. Repeat interviews	Were repeat interviews carried out? If yes, how many?	No
19. Audio/visual recording	Did the research use audio or visual recording to collect the data?	Yes, in Methods
20. Field notes	Were field notes made during and/or after the interview or focus group?	Field notes were made.
21. Duration	What was the duration of the inter views or focus group?	Between 45 – 60 min (in Methods).
22. Data saturation	Was data saturation discussed?	Yes (in Methods)
23. Transcripts returned	Were transcripts returned to participants for comment and/or correction?	Transcripts were not returned to participants.
**Domain 3: analysis and findings**		
Data analysis		
24. Number of data coders	How many data coders coded the data?	2 (in data analysis).

25. Description of the coding tree	Did authors provide a description of the coding tree?	Yes, but not presented in the manuscript.
26. Derivation of themes	Were themes identified in advance or derived from the data?	Themes were derived from the data and classified according to the COM-B model (in data analysis).
27. Software	What software, if applicable, was used to manage the data?	NVivo was used (in data analysis).
28. Participant checking	Did participants provide feedback on the findings?	No, participants were not provided with the findings.
Reporting		
29. Quotations presented	Were participant quotations presented to illustrate the themes/findings? Was each quotation identified? e.g. participant number	Quotations were presented and participant number provided (in Table 2).
30. Data and findings consistent	Was there consistency between the data presented and the findings?	Consistency was checked (in Results).
31. Clarity of major themes	Were major themes clearly presented in the findings?	Major themes were clearly presented (in Results and Table 2).
32. Clarity of minor themes	Is there a description of diverse cases or discussion of minor themes?	Minor themes and diverse cases were described (in Results).
